# Complete Genome Sequences of the Historical *Legionella pneumophila* Strains OLDA and Pontiac

**DOI:** 10.1128/genomeA.00866-16

**Published:** 2016-08-25

**Authors:** Jeffrey W. Mercante, Shatavia S. Morrison, Brian H. Raphael, Jonas M. Winchell

**Affiliations:** Division of Bacterial Diseases, National Center for Immunization and Respiratory Diseases, Centers for Disease Control and Prevention, Atlanta, Georgia, USA

## Abstract

Here, we report the complete genome sequences of *Legionella pneumophila* serogroup 1 strains OLDA and Pontiac, which predate the 1976 Philadelphia Legionnaires’ disease outbreak. Strain OLDA was isolated in 1947 from an apparent sporadic case, and strain Pontiac caused an explosive outbreak at a Michigan health department in 1968.

## GENOME ANNOUNCEMENT

Legionnaires’ disease (LD) is a severe and sometimes fatal bacterial pneumonia caused by a growing list of *Legionella* spp., the most clinically relevant is *L. pneumophila*. One of the first high-profile LD outbreaks occurred in 1976 at an American Veterans’ convention in Philadelphia (1). Eight years prior to the Philadelphia outbreak, a large epidemic of febrile, nonpneumonic disease, termed “Pontiac Fever,” sickened 144 individuals at the Oakland County health department in Pontiac, Michigan (2). The cause of this illness was identified in 1977 when CDC researchers successfully isolated *L. pneumophila* serogroup 1 (sg1) from previously frozen guinea pig tissues experimentally inoculated with the Pontiac agent in 1968 (3). The “rickettsia-like” organism, OLDA, among the earliest cultured legionellae, was originally isolated in 1947 (4) from an apparent sporadic LD case; in 1977, this organism was identified as the same species and serogroup as the Philadelphia outbreak bacterium (5). Both strains were recently propagated from frozen stocks in the CDC archival collection with storage dates of 25 July 1978 for strain OLDA and 10 April 1978 for strain Pontiac.

Pacific Biosciences RSII-compatible (Menlo Park, CA, USA) long-insert DNA libraries were constructed for both strains according to the manufacturer’s 10-kb protocol (PN100-286-100-04) and sequenced on single SMRT cells using 240-min movies. Assembly was performed with the Hierarchical Genome Assembly Process 3 (HGAP3) (6) through the PacBio SMRT Analysis System at ~85× coverage using 80,689 and 70,696 reads for the OLDA and Pontiac genomes, respectively. Overlapping contig ends were identified with Gepard version 1.3 (7) and trimmed to produce closed, circular genomes; 2,541,000 (OLDA) and 2,869,376 (Pontiac) paired-end 250-bp Illumina MiSeq (San Diego, CA, USA) reads were mapped to the PacBio assemblies to verify nucleotide accuracy >99.99%. The main OLDA genome is 3,486,082 bp with one circular plasmid, pLP3 (8) (G+C of 37.5%), of 129,883 bp, while strain Pontiac is 3,544,954 bp without extrachromosomal elements; both genomes have a G+C content of 38.4%. The NCBI Prokaryotic Genome Annotation Pipeline (PGAP) (9) identified 3,135 and 3,084 predicted protein coding genes for OLDA and Pontiac, respectively, and nine rRNAs, 43 tRNAs, and four ncRNAs in both strains; strain OLDA harbored a single CRISPR array. Genome analyses with Geneious version 9 (10) revealed that the previously described ~30-kb unstable genetic element (11) was absent from strain OLDA, but a pP36-like element (12) is maintained. A deletion of the *lag-1* O-acetyltransferase also corroborates its monoclonal antibody-2 (mAb2 = mAb3/1) negative phenotype (13, 14). Strain OLDA is sequence type 1 (ST1) (15) and highly similar (≥98.5%) to the ST1 *L. pneumophila* reference strain Paris (NC_006368) (16). Strain Pontiac (ST62) is 93.4% identical to the *L. pneumophila* reference strain L10-023 (NC_002942.5) of the same sequence type, but lacks a 70.5-kb genomic island (position 3,042,379), and exhibits a genomic rearrangement near a *trb/vir* conjugal transfer locus (at position ~2,729,000).

The complete genomes of strains OLDA and Pontiac, among the oldest known clinical and environmental *L. pneumophila* isolates, respectively, will be valuable as reference sequences as we attempt to understand the diversity of this species and its public health importance.

### Accession number(s).

The whole-genome sequences described here have been deposited at NCBI/GenBank under the accession numbers CP016030 (OLDA chromosome), CP016031 (OLDA plasmid), and CP016029 (Pontiac).
